# A novel fluorescence immunoassay for the quantitative detection of HPV16 L1 antibodies in human serum samples using ZnCdSe/ZnS quantum dot-labeled antibodies

**DOI:** 10.1128/spectrum.01843-24

**Published:** 2025-04-08

**Authors:** Aiping Wang, Cheng Xin, Zhuting Chen, Jingming Zhou, Yumei Chen, Yankai Liu, Hongliang Liu, Chao Liang, Xifang Zhu, Yanhua Qi, Gaiping Zhang

**Affiliations:** 1 Longhu Laboratory 12636 , Zhengzhou, China; 2 School of Life Sciences, Zhengzhou University 693032 , Zhengzhou, Henan, China; 3 Henan Key Laboratory of Immunobiology, Zhengzhou, China; 4 School of Advanced Agricultural Sciences, Peking University 12465 , Beijing, China; 5 College of Veterinary Medicine, Henan Agricultural University 70573 , Zhengzhou, China; Petrified Bugs LLC, Miami, Florida, USA

**Keywords:** HPV16-L1, antibody, QDs-B-ELISA

## Abstract

Human papillomavirus type 16 (HPV16) is a high-risk virus linked to cervical cancer, primarily through its oncogenic proteins E6 and E7. The HPV16-L1 protein, the major capsid component, plays a key role in capsid formation and immune response. Monitoring anti-HPV16-L1 antibodies in serum is crucial for understanding infection dynamics and vaccine efficacy. This study aimed to develop a novel quantum dot-labeled blocking enzyme-linked immunosorbent assay (QDs-B-ELISA) for the quantitative detection of anti-HPV16-L1 antibodies. Monoclonal antibodies were produced and characterized against HPV16-L1 virus-like particles. A QDs-B-ELISA method was developed based on these antibodies and evaluated using 199 serum samples with previously established HPV16 status (“known” samples) and 170 serum samples with unknown HPV16 status at the time of testing (“unknown” samples). The diagnostic accuracy, sensitivity, specificity, and quantitative detection range of the QDs-B-ELISA were assessed and compared with commercial ELISA kits. The established QDs-B-ELISA exhibited high diagnostic accuracy (area under the curve, AUC = 0.9945), sensitivity (95.83%), and specificity (96.85%) for known serum samples. The lower limit of HPV16 antibody concentration detected by QDs-B-ELISA (0.0875 IU/mL) was considerably lower than that of the commercial ELISA kit, the Human Anti-HPV16-L1 Antibody (IgG) ELISA Kit (LS-F10262-1, Lsbio) (0.35 IU/mL), with a quantitative detection range of 13–1,737.8 IU/mL. When analyzing unknown human serum samples, the QDs-B-ELISA demonstrated a 97.06% agreement with commercial kits, and both inter-assay and intra-assay coefficients of variation were below 10%. The QDs-B-ELISA demonstrated high stability, sensitivity, and specificity, offering a valuable tool for surveillance and epidemiological studies of HPV16 infection.

**IMPORTANCE:**

This study introduces a novel quantum dot-labeled blocking enzyme-linked immunosorbent assay for detecting anti-HPV16-L1 antibodies, offering superior sensitivity and specificity compared to conventional methods. The improved performance enables more accurate HPV16 surveillance, epidemiological studies, and vaccine efficacy monitoring. This advancement may enhance early detection and risk assessment of HPV16 infections.

## INTRODUCTION

Cervical cancer, primarily caused by high-risk human papillomavirus (HPV) infections, remains a significant global health concern (1), particularly in low- and middle-income countries where access to screening and prevention programs is limited (2). HPV is a small, non-enveloped, double-stranded DNA virus that infects epithelial cells, potentially triggering cellular proliferation and malignant transformation (3). Among the HPV types, HPV16 is most frequently associated with cervical cancer, and its major capsid protein, L1 (HPV16-L1), serves both as a critical component for vaccine development and as a biomarker for early infection. Early detection of HPV16-L1 is pivotal for timely intervention and prevention of disease progression, highlighting the need for accurate and efficient diagnostic tools.

Currently, nucleic acid-based detection methods, such as polymerase chain reaction (PCR) (4), loop-mediated isothermal amplification (5), and rolling circle amplification (6), are widely used to detect HPV infections. Additionally, ThinPrep cytology provides insight into early cellular changes associated with cervical cancer. These techniques have substantially reduced the global burden of cervical cancer by enabling early diagnosis. However, their widespread use is hindered by high equipment costs, long detection times, and the need for technical expertise, limiting their accessibility in some settings.

To complement these diagnostic methods, serological assays have garnered interest for their non-invasive approach, particularly for monitoring vaccine-induced immune responses. Although their application for primary screening remains limited, serological assays are valuable for evaluating immune responses in vaccinated individuals. Among serological detection methods, enzyme-linked immunosorbent assay (ELISA) is one of the most commonly used and well-established techniques. ELISA is based on the specific interaction between antigens and antibodies, with quantitative analysis achieved through detectable signals generated by enzyme-labeled markers. It is widely applied in the detection of both antibodies and antigens, offering high sensitivity, good reproducibility, and ease of standardization. However, it may encounter challenges such as non-specific binding when applied to certain complex samples. In ELISA, blocking ELISAs (B-ELISAs) are also frequently employed, which measure antibodies that inhibit the binding of monoclonal antibodies to viral antigens and have demonstrated high sensitivity and specificity.

Several serological assays have been developed for HPV-specific antibody detection, including competitive Luminex immunoassay (cLIA), virus-like particle multiplex immunoassay (VLP-MIA), M4ELISA, and M9ELISA, as well as high-throughput pseudovirion-based neutralization assays (PBNAs) (7–10). Organizations such as the Centers for Disease Control and Prevention and the HPV Serology Laboratory rely on these methods to monitor HPV-related infections and vaccination outcomes. Additionally, ELISA and antibody-blocking ELISA have also been widely applied for HPV detection and successfully employed for detecting a range of viruses in both humans and animals, including Zika virus (11), influenza virus (12), porcine delta-coronavirus (13), African swine fever virus (14–16), foot-and-mouth disease virus (17, 18), and SARS-CoV-2 (19). However, conventional ELISA or antibody-blocking ELISA exhibits limitations such as suboptimal sensitivity, photostability issues, and fluorescence quenching, which can impact diagnostic accuracy.

Quantum dots (QDs) have emerged as a novel fluorescent probe material that addresses these limitations. QDs exhibit unique optical properties, including narrow emission peaks, large Stokes shifts, and high quantum yields, which enhance photostability and detection sensitivity. These properties address key challenges in conventional ELISA, such as fluorescence quenching and photostability issues, making QD-based assays a promising alternative to traditional fluorescent dyes. By integrating QDs with antibodies, advanced immunoassay platforms can achieve improved signal-to-noise ratios and broader detection ranges.

In this study, we developed a ZnCdSe/ZnS QDs-labeled antibody-based blocking-ELISA (QDs-B-ELISA) to quantitatively detect anti-HPV16-L1 antibodies in human serum. The use of HPV16-L1 virus-like particles (VLPs) as the antigen enabled specific detection, and the QDs-based platform offered improved sensitivity compared to traditional ELISAs. Our QDs-B-ELISA provides a rapid, sensitive, and cost-effective tool for monitoring HPV16-related infections and vaccine-induced immune responses, supporting disease management and prevention strategies.

## MATERIALS AND METHODS

### Construction of Hpv16-L1 expression vector

The HPV16-L1 nucleic acid sequence (GenBank: KU721788.1) was integrated into the pET-SUMO vector using *Bsa*I and *Xho*I restriction endonucleases (NEB, New England Biolabs, USA) and T4 DNA ligase ( TakaRa Biotechnology Co., Ltd., Dalian, China). The ligation product was transformed into DH5α-competent cells, plated on ampicillin-containing agar, and incubated overnight at 37°C. Single clones were PCR verified (primers: 5′-ATGTCTTTGTGGTTGCCATCTGA-3′ and 5′-CTATTACAACTTTCTCTTCTTTCT-3′), and plasmids were enzyme digestion verified (*Bsa*I and *Xho*I). Sequencing-confirmed plasmids were transformed into BL21 cells for further experiments.

### Expression and purification of recombinant HPV16-L1 protein

The BL21 bacterial strain was used for the expression of the HPV16-L1 protein, induced with 1 mM IPTG at a shaking temperature of 18°C. Protein expression was analyzed through SDS-PAGE. Under these conditions, bacterial cells were harvested and resuspended in PBS containing 1% Phenylmethylsulfonyl Fluoride (PMSF), followed by sonication on ice to lyse the cells. The supernatant was filtered through a 0.22 µm membrane, and the recombinant protein was purified using Ni-NTA resin. The purity and identity of the protein were assessed by SDS-PAGE and immunoblotting with anti-His monoclonal antibody (20).

### Western blot assay

Protein samples were separated by SDS-PAGE and transferred onto a PVDF membrane. The membrane was blocked with 5% non-fat milk in PBST, incubated with a specific primary antibody at 37°C for 1 h, washed, and then incubated with an HRP-labeled secondary antibody at 37°C for 1 h. After washing, a chemiluminescent substrate was added, and the target protein expression was detected using a Tanon imaging system.

### Assembly and morphological analysis of HPV16-L1 VLPs

The SUMO tag was removed from HPV16-L1 protein by SUMO protease digestion and gel filtration chromatography. DTT (10 mmol/L) was added to the tag-free protein and incubated at 4°C for 2 h. The sample was purified using Sephadex G-25 gel filtration chromatography, eluted with assembly buffer, and incubated at 4°C for 72 h to assemble VLPs. The VLPs were filtered (0.22 µm), and their particle size was analyzed using a Malvern Zetasizer Nano ZS90 and Zetasizer Software.

### Animal immunization strategy and screening of positive hybridoma cells

Female BALB/c mice (6–8 weeks) were immunized subcutaneously with 50 µg/200 µL of HPV16-L1 VLPs mixed with Freund’s adjuvant (complete for initial and incomplete for boosters) every 2 weeks. Serum antibody titers were measured 1 week post-third immunization, and the highest-titer mouse received an intraperitoneal booster. Three days later, spleen cells were fused with SP2/0 cells (8:1) and seeded in HAT medium for hybridoma screening. Positive hybridomas were detected by indirect ELISA and purified through three rounds of limited dilution to obtain a monoclonal antibody cell line, which was expanded and cryopreserved.

### Preparation, purification, and subtype identification of monoclonal antibodies

Healthy multiparous BALB/c mice were injected with Freund’s incomplete adjuvant (500 µL, i.p.). Expanded hybridoma cells (2 × 10^6^ cells/mL in serum-free 1640 medium, 500 µL) were injected i.p. into mice with slight abdominal swelling. Ascites were collected, centrifuged (6,000 rpm for 10 min), and purified using the caprylic acid-ammonium sulfate method. Caprylic acid was added, the clear liquid was collected, filtered, and adjusted to pH 7.4 with 10× PBS. Subsequently, this solution was diluted with water to achieve a final concentration of 1× PBS. The mixture was cooled to 4°C. Ammonium sulfate (50% saturation) was added, stirred, and centrifuged. The precipitate was resuspended in PBS (1/3 volume), dialyzed at 4°C, centrifuged, aliquoted, and labeled. Antibody subtypes were identified (Proteintech kit) and stored at −80°C.

### Indirect enzyme-linked immunosorbent assay

HPV16-L1 VLPs in carbonate buffer solution (CBS) were added to 96-well plates (1.2 µg/mL; 100 µL/well), incubated overnight at 4°C, and blocked with 5% skimmed milk in PBST for 45 min at 37°C. After washing the wells with PBST, a specific primary monoclonal antibody against HPV16-L1, which was generated and screened using hybridoma technology, was added and incubated for 1 h at 37°C and washed again. Diluted Goat Anti-Mouse IgG Fab-HRP (Abbkine) was added and incubated at 37°C for 1 h. After five washes, TMB solution (Beyotime) was added (100 µL/well) for 10 min, and the reaction was terminated with 2 mol/L sulfuric acid. The optical density (OD) values at 450 nm were measured.

### Titer and affinity determination of HPV16-L1 VLP monoclonal antibodies

The antigen was coated at 1 and 2 µg/mL in CBS buffer (100 µL/well) overnight at 4°C. Monoclonal antibody affinity was determined by indirect ELISA at different coating concentrations. Two curves were plotted: antibody concentration vs OD_450nm_ and 1/concentration vs 1/OD_450nm_. Antibody concentrations corresponding to 50% OD_450nm_ were calculated and converted to molar concentrations. The affinity constant (*K*_aff_) was calculated using *K*_aff_ = (*n* − 1)/2(*n*[Ab′]*t* − [Ab]*t*), where *n* = [Ag]*t*/[Ag′]*t* and [Ab]*t* and [Ab′]*t* are molar concentrations at 50% OD_450nm_.

### Standard sera and test samples

The reliability of the blocking ELISA was validated using HPV16 antibody-positive and antibody-negative standard sera from the China National Institutes for Food and Drug Control (CNIFDC). A total of 199 inactivated test samples (72 HPV16 antibody negative and 127 HPV antibody positive) were analyzed. The HPV16 antibody-positive standard serum had a concentration of 28 IU/mL (5.6 IU/vial). Test serum samples were obtained from patients at Henan Cancer Hospital, China.

### Screening of MAbs suitable for QDs-B-ELISA

To screen mAbs for QDs-B-ELISA, titers were compared using HPV16-L1 VLPs (2 µg/mL) as coating antigens. Plates were coated, blocked, and incubated with serially diluted mAbs. HRP-conjugated goat anti-mouse IgG was added, and titers were compared using OD_450nm_ of 1.0 as the reference. HRP-labeled mAbs were prepared by coupling HRP to purified mAbs, precipitating with ammonium sulfate, and dialyzing. Titers were determined by direct ELISA using OD_450nm_ of 2.0 as the reference. The highest-titer HRP-mAb was selected and tested in B-ELISA with positive and negative standard sera. The mAb with the highest blocking rate for positive sera and lowest for negative sera was considered optimal.

### Preparation of immunolabeling probes based on QDs

Quantum dot-labeled fluorescent probes were prepared using the EDC method (21). Water-soluble QDs (ZnCdSe/ZnS, QDs-COOH, 8 mM, *λ*_max_ = 605 nm) were mixed with EDC (1 mg/mL in PBS) at a 1:2,000 molar ratio and incubated at 25°C for 30 min (22). 1A8 mAb (1 mg/mL in PBS) was added (QDs:mAb = 1:5) and reacted under sealed conditions. BSA (1 mg/mL, 20 µL) was added to block free QDs carboxyl sites (25°C, 30 min). The final probe was stored at 4°C, protected from light (23, 24).

### Characterization of QDs and fluorescent probe

The fluorescent probe was characterized using methods from previous studies (25). UV-visible absorption spectra were obtained using Nanodrop 2000c, and fluorescence spectra of QDs coupled with 1A8 antibody and pure QDs were determined using multifunctional enzyme markers. Agarose gel electrophoresis and SDS-PAGE were performed. Successful conjugation was confirmed by analyzing particle size and zeta potential using DLS with a Malvern particle size analyzer.

### Development of the quantum dot-labeled B-ELISA

HPV16-L1 VLP antigen was coated onto 96-well plates and blocked with 1% BSA. Standard HPV16 antibody-positive and negative control sera were added to odd and even wells, respectively, and incubated at 37°C for 30 min. Pre-screened optimal QDs-mAb was serially diluted and added to the wells, then incubated at 37°C for 30 min. Fluorescence intensity at 450 nm was measured, and the blocking rate of the positive control serum against QDs-mAb was calculated. The optimal QDs-mAb concentration was selected based on the strongest blocking ability for positive serum and the weakest for negative serum. The QDs-B-ELISA standard curve was established using optimal conditions, with *F* and *F*_0_ representing fluorescence intensity with and without human HPV16 standard positive serum, and the horizontal coordinate representing antibody concentration in the standard positive serum.

### Optimized quantum dot-labeled B-ELISA methods

HPV16-L1 VLPs were coated on 96-well plates at various dilutions and incubated at 37°C for 1–2 h or 4°C for 12 h. Plates were washed with PBST, blocked with 5% skimmed milk at 37°C for 30–120 min, and washed again. Diluted control and assay sera were added, incubated at 37°C for 30 min–2 h, and washed. QDs-labeled antibody was added, incubated at 37°C for 30 min–2 h, and washed thoroughly. Fluorescence intensity was measured using a multifunctional microplate reader. Experimental conditions, including coating concentration, antibody dilution, incubation times, and serum dilution, were optimized. The percentage of inhibition (PI) was calculated for each serum sample using the formula: PI = ([fluorescence intensity of negative control **–** fluorescence intensity of positive control]**/**fluorescence intensity of negative control) × 100% (26, 27).

### Determination of cutoff value, diagnostic sensitivity, and specificity

To calculate the optimal critical value and the associated diagnostic sensitivity and specificity, standard serum and test samples were assayed. The assay data were analyzed by ROC through GraphPad software, and the associated data were calculated (14, 28, 29).

### Assessment of specificity, sensitivity, and reproducibility of the QDs-B-ELISA

Specificity was confirmed by testing eight polyclonal antisera against other HPV subtypes using the developed QDs-B-ELISA. Reproducibility was evaluated by running 10 control sera (five positive and five negative). Mean, standard deviation, and coefficient of variation were calculated using SPSS software version 26.0. Under optimal detection conditions (Tables 1 and 2), the method was applied to serially diluted standard HPV16-positive sera, keeping other conditions constant. The inhibition rate was calculated at different dilutions using the optimal cutoff value as the threshold. The maximum dilution factor exceeding the cutoff value was considered the detection limit.

**TABLE 1:** Exploring optimal HPV16-L1 VLPs coating concentration and HRP-1A8 mAb dilution

Standard serum	HRP-1A8 mAb dilution	Antigen dilution						
		1:100	1:200	1:400	1:800^*a*^	1:1,600	1:3,200	1:6,400
HPV16 antibody-negative standard serum	1:1,000	1.454	1.186	0.948	0.626	0.527	0.474	0.383
	1:2,000	1.347	1.132	0.708	0.576	0.435	0.374	0.336
	1:3,000	1.126	0.965	0.664	0.426	0.362	0.265	0.215
	1:4,000	0.846	0.728	0.565	0.383	0.357	0.253	0.210
	**1:5,000^*a*^**	0.752	0.665	0.468	**0.268^*a*^**	0.245	0.202	0.174
	1:6,000	0.679	0.538	0.352	0.276	0.258	0.183	0.126
	1:7,000	0.566	0.362	0.259	0.252	0.193	0.184	0.142
	1:8,000	0.473	0.358	0.364	0.238	0.176	0.152	0.141
	1:9,000	0.461	0.369	0.384	0.228	0.184	0.174	0.135
	1:10,000	0.394	0.372	0.263	0.212	0.194	0.174	0.153
HPV16 antibody-positive standard serum	1:1,000	2.414	2.367	1.984	1.674	1.128	0.864	0.793
	1:2,000	2.584	2.126	1.758	1.529	0.965	0.765	0.568
	1:3,000	2.055	1.968	1.553	1.338	0.785	0.663	0.663
	1:4,000	1.877	1.664	1.368	1.148	0.668	0.558	0.465
	**1:5,000^*a*^**	1.745	1.527	1.384	**1.021^*a*^**	0.827	0.592	0.425
	1:6,000	1.533	1.368	1.065	0.845	0.763	0.465	0.375
	1:7,000	1.358	1.165	0.968	0.757	0.568	0.458	0.358
	1:8,000	1.154	1.068	0.736	0.562	0.436	0.386	0.393
	1:9,000	0.938	0.853	0.683	0.558	0.485	0.336	0.285
	1:10,000	0.655	0.568	0.557	0.463	0.338	0.236	0.221
HPV16 antibody-negative standard serum/HPV16 antibody-positive standard serum = N/P	1:1,000	1.660	1.996	2.093	2.674	2.140	1.823	2.070
	1:2,000	1.918	1.878	2.483	2.655	2.218	2.045	1.690
	1:3,000	1.825	2.039	2.339	3.141	2.169	2.502	3.084
	1:4,000	2.219	2.286	2.421	2.997	1.871	2.206	2.214
	**1:5,000^*a*^**	2.320	2.296	2.957	**3.810^*a*^**	3.376	2.931	2.443
	1:6,000	2.258	2.543	3.026	3.062	2.957	2.541	2.976
	1:7,000	2.399	3.218	3.737	3.004	2.943	2.489	2.521
	1:8,000	2.440	2.983	2.022	2.361	2.477	2.539	2.787
	1:9,000	2.035	2.312	1.779	2.447	2.636	1.931	2.111
	1:10,000	1.662	1.527	2.118	2.184	1.742	1.356	1.444

^*a*^ Bold values in the table represents the optimal conditions tested.

**TABLE 2:** Optimization of detection conditions

Optimization categories	Detection conditions	HPV16 antibody-positive standard serum (OD_450nm_)	HPV16 antibody-negative standard serum (OD_450nm_)	N/P
Antigen coating time	37°C for 1 h	0.215	0.946	4.400
	**37°C for 2 h^*a*^**	0.247	1.114	**4.510^*a*^**
	4°C for 12 h	0.287	1.135	3.955
Blocking time	30 min	0.325	1.246	3.834
	**45 min^*a*^**	0.311	1.379	**4.434^*a*^**
	1 h	0.292	1.158	3.966
	2 h	0.327	1.263	3.862
Serum dilutions	1:5	0.425	1.352	3.181
	1:10	0.349	1.337	3.831
	1:20	0.364	1.252	3.440
	1:40	0.228	1.257	5.513
	**1:80^*a*^**	0.257	1.442	**5.611^*a*^**
	1:160	0.516	1.163	2.254
	1:320	0.748	1.325	1.771
	1:640	0.964	1.272	1.320
Serum incubation time	30 min	0.284	0.943	3.320
	45 min	0.264	1.124	4.258
	**1 h^*a*^**	0.258	1.264	**4.899^*a*^**
	2 h	0.236	1.052	4.458
Monoclonal antibody incubation time	30 min	0.198	0.824	4.162
	45 min	0.239	1.052	4.402
	**1 h^*a*^**	0.174	1.053	**6.052^*a*^**
	2 h	0.253	0.943	3.727
TMP color development time	5	0.184	0.695	3.777
	**10^*a*^**	0.236	1.052	**4.458^*a*^**
	15	0.340	1.243	3.656
	20	0.451	1.632	3.619
	25	0.432	2.136	4.944

^*a*^ Bold values in the table represents the optimal conditions tested.

### Statistical analysis of data

Unless otherwise specified, all statistical analyses and assessments of significant differences were conducted using GraphPad Prism software, employing *t*-tests. The results of the significant difference analysis are presented as follows: ns, *P* > 0.05, no significant difference; *P ≤ 0.05, significant difference; **P ≤ 0.01, highly significant difference; and ***P ≤ 0.001, extremely significant difference between the means of the two data sets.

## RESULTS

### Preparation of antigens and identification of activity

**Fig 1:**
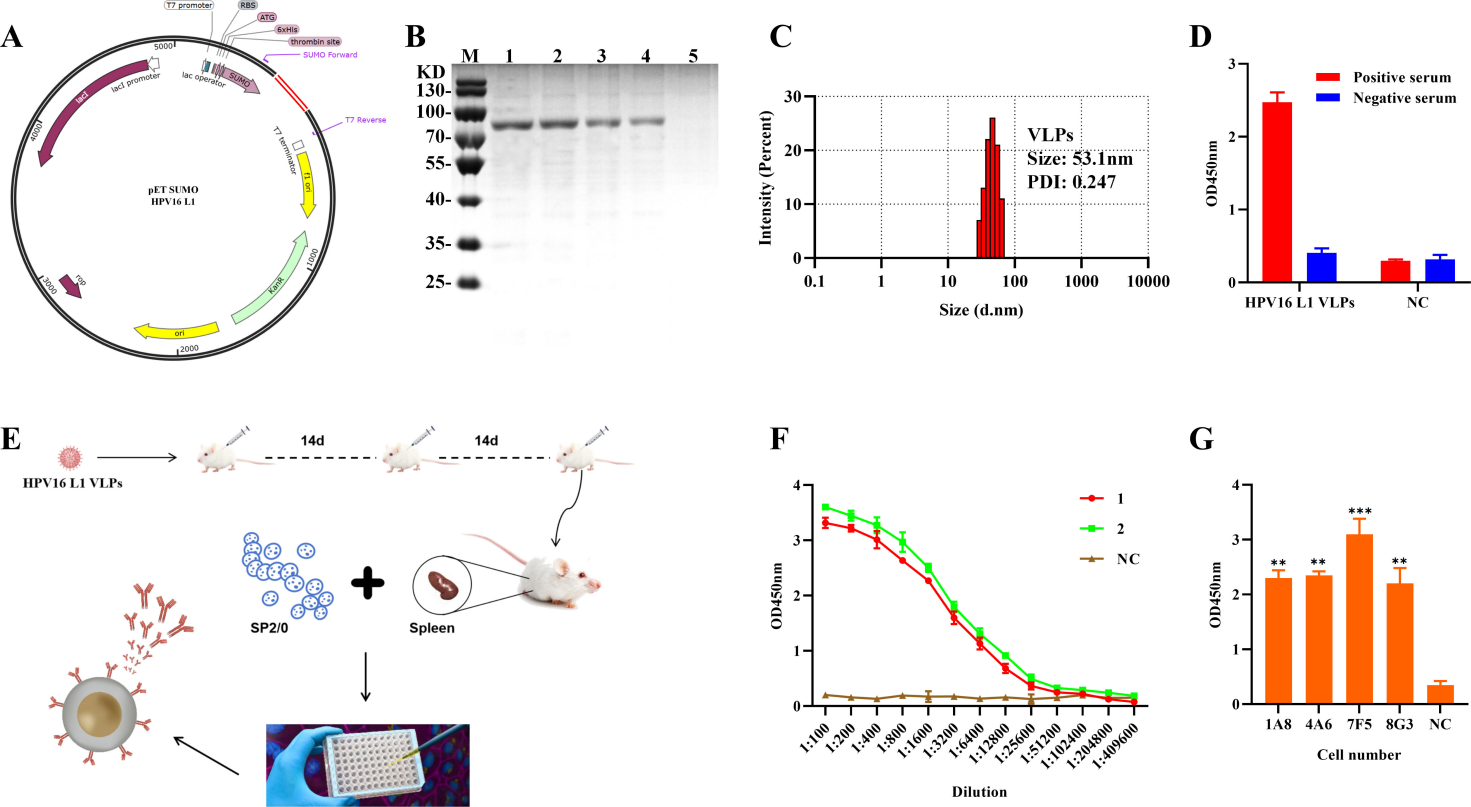
Construction of HPV16-L1 VLPs and immunization of mice. (**A**) Schematic representation of the pET-SUMO-HPV16-L1 vector construction. (**B**) Western blot analysis confirmed the binding of anti-His tag antibodies to the protein at the expected position, verifying that the protein detected at this position was HPV16-L1. Lane M contains the protein marker, while lanes 1, 2, 3, and 4 contain the SUMO-HPV16-L1 recombinant protein. Lane 5 serves as a negative control, containing BSA without a His tag. (**C**) Particle size analysis demonstrates the dynamic light scattering of the protein sample 72 h after self-assembly. The results indicate the presence of abundant spherical particles around 50 nm, consistent with the size of HPV, suggesting that the prokaryotically expressed HPV16-L1 protein can self-assemble into native-like VLPs *in vitro*. (**D**) ELISA results show that the obtained HPV16-L1 VLPs react with standard sera positive for HPV16 antibodies (28 IU/mL). In contrast, no reactivity was observed with standard sera negative for HPV16-L1 antibodies. This finding further confirms the biological activity of the protein. (**E**) Schematic representation of the process from multi-site subcutaneous immunization of BALB/c mice to the generation of monoclonal antibodies against HPV16-L1 VLPs. (**F**) Titration of tail bleeding from BALB/c mice after the third immunization. (**G**) Four hybridoma cell lines targeting HPV16-L1 VLPs were obtained through cell fusion.

The HPV16-L1 gene fragment was obtained from NCBI and used to construct the pET-SUMO recombinant protein for expression in BL21 (Fig. 1A). Western blot analysis using an anti-His monoclonal antibody confirmed the identity of the recombinant protein (SUMO-HPV16-L1, 78.73 kDa), with a clear band at the predicted position in an SDS-PAGE-stained gel (Fig. 1B). The assembled HPV16 VLPs were analyzed using a Malvern Zetasizer Nano ZS90 particle size analyzer, revealing abundant spherical particles around 50 nm in size, consistent with the size of HPV (Fig. 1C). This indicates that the prokaryotically expressed HPV16-L1 protein could self-assemble into native VLPs *in vitro*. Furthermore, the purified HPV16 VLPs reacted with the standard positive serum in an indirect ELISA assay, demonstrating the biological activity of the HPV16-L1 protein obtained using *Escherichia coli* (Fig. 1D).

### Preparation of monoclonal antibodies against HPV16-L1 VLPs

**Fig 2:**
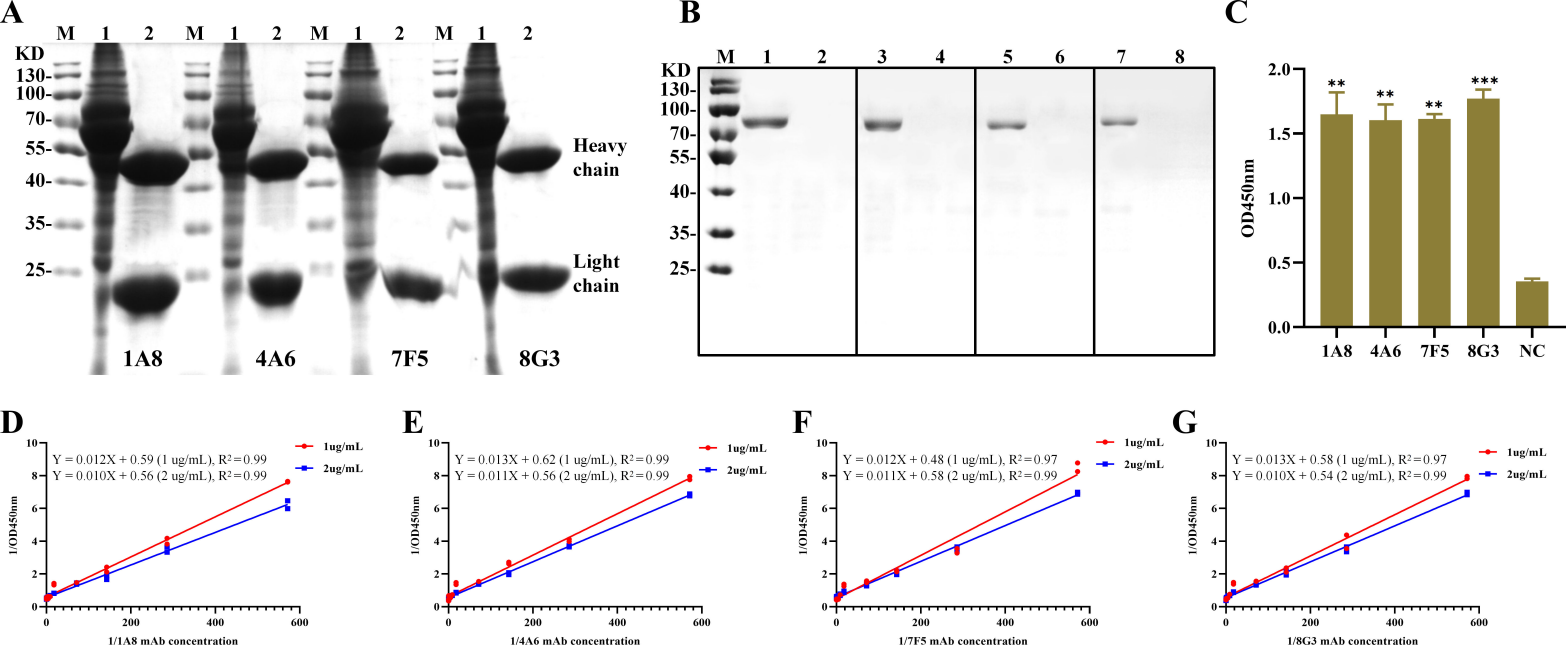
Purification, identification, and blocking effect detection of monoclonal antibodies. (**A**) Monoclonal antibodies were purified using the octanoic acid-ammonium sulfate method. M represents the protein marker, lane 1 contains the antibody before purification (ascites), and lane 2 contains the purified monoclonal antibody, with heavy and light chains appearing around 55 and 25 kDa, respectively. (**B**) Western blot analysis was employed to verify the reactivity of the four generated monoclonal antibodies against the HPV16-L1 protein. The protein marker is labeled as M. Lanes 1, 3, 5, and 7 contain the recombinant HPV16-L1 protein, while lanes 2, 4, 6, and 8 contain the SUMO-tagged protein. The membrane was probed with the following primary antibodies: lanes 1 and 2 with 1A8, lanes 3 and 4 with 4A6, lanes 5 and 6 with 7F5, and lanes 7 and 8 with 8G3. The secondary antibody used was an HRP-labeled goat anti-mouse antibody. (**C**) ELISA results demonstrate the reactivity of the four monoclonal antibodies against HPV16-L1 VLPs, while NC represents the SUMO-tagged protein used as a negative control. (**D–G**) Fitting curves for the affinity determination of the following monoclonal antibodies to HPV16-L1 VLPs.

Monoclonal antibodies were generated by immunizing mice with HPV16-L1 VLPs (Fig. 1E). One week after the third immunization, the tail bleed titers of mice 1 and 2 reached 1:10E4 and 1:20E4, respectively (Fig. 1F). Mouse 2 was selected for cell fusion due to its higher titer. Following fusion, monoclonal antibodies with high specificity and antagonism were screened by HPV16-L1 VLPs indirect ELISA, resulting in the identification of positive hybridoma cells 1A8, A46, 7F5, and 8G3 (Fig. 1G). High-purity monoclonal antibodies 1A8, A46, 7F5, and 8G3 were obtained from mouse ascites using the octanoic acid-ammonium sulfate assay (Fig. 2A). Isotype characterization using the mouse Ig Isoform kit revealed that all four monoclonal antibodies were IgG1 with a kappa light chain.

### Characterization of monoclonal antibodies and evaluation of blocking effects

**Fig 3:**
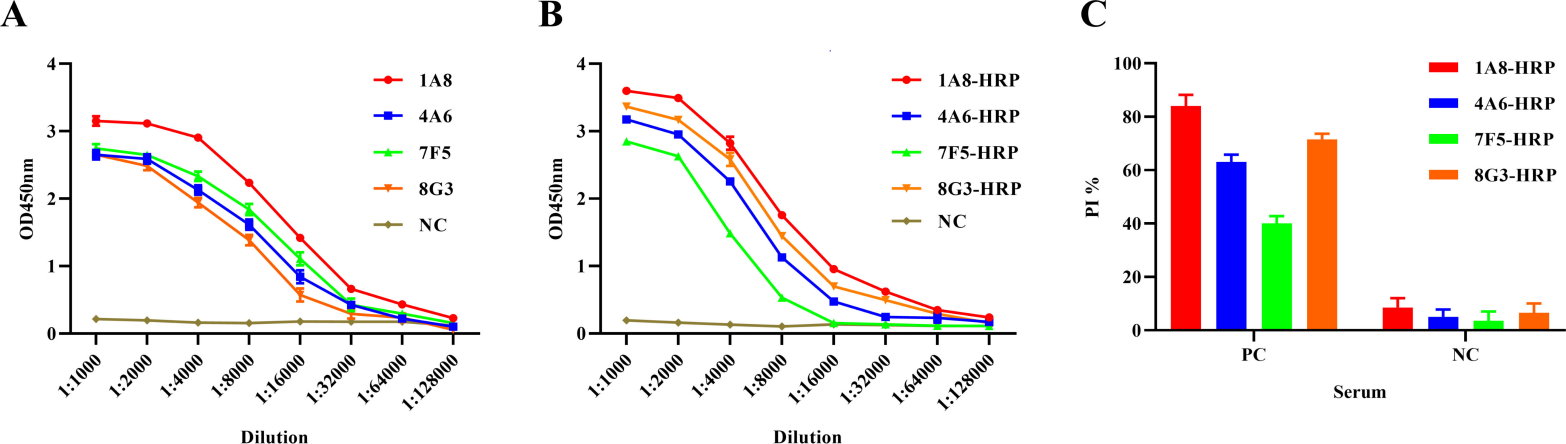
Evaluation of monoclonal antibody titers and blocking effects using various ELISA methods. (**A**) Indirect ELISA was used to determine the titer of the purified monoclonal antibodies. (**B**) Direct ELISA was employed to assess the titer of the HRP-labeled HPV16-L1 monoclonal antibodies. (**C**) The B-ELISA method was utilized to compare the blocking effect of the four monoclonal antibodies against standard sera positive and negative for HPV16 antibodies.

Figure 2B and C confirms the efficacy of four monoclonal antibodies against HPV16-L1 VLPs, as determined by Western blot and indirect ELISA. Antigen-antibody affinity detection revealed KAFF constants of 3.3 × 10^9^ L/mol, 2.6 × 10^9^ L/mol, 2.3 × 10^9^ L/mol, and 2.5 × 10^9^ L/mol for 1A8, A46, 7F5, and 8G3, respectively (Fig. 2D through G). Indirect ELISA titer detection showed that 1A8 reached 1:128,000, while 4F6, 7F5, and 8G3 reached 1:64,000 (Fig. 3A). Direct ELISA using HRP-labeled antibodies demonstrated that 1A8 and 8G3 reached 1:64,000, 4A6 reached 1:32,000, and 7F5 reached 1:16,000 (Fig. 3B). Blocking effect evaluation revealed that 1A8 had the highest blocking rate at 80%, followed by 4A6 and 8G3 at 60%, and 7F5 at 40%. Standard negative sera had blocking rates below 20% (Fig. 3C). The 1A8 antibody, exhibiting the best evaluation data, was selected to establish the QDs-B-ELISA method.

### Evaluation of fluorescent probes for single QDs

**Fig 4:**
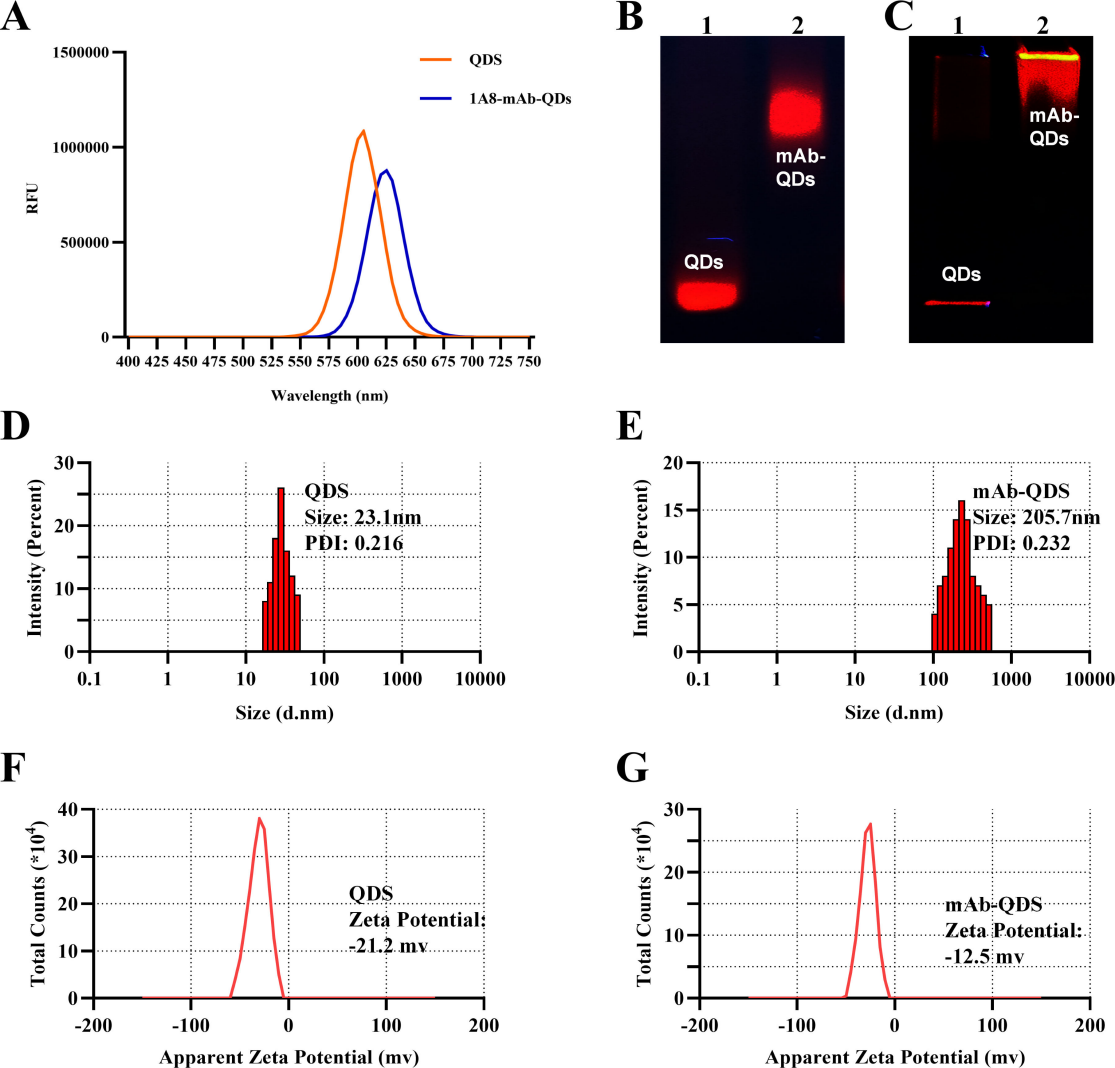
Characterization of conjugated quantum dots. (**A**) Fluorescence emission spectra of quantum dots and mAb-quantum dot conjugates. (**B**) Agarose gel electrophoresis of quantum dots (lane 1) and mAb-quantum dot conjugates (lane 2). (**C**) SDS-PAGE analysis of quantum dots (lane 1) and mAb-quantum dot conjugates (lane 2). Particle size distribution of quantum dots (**D**) and mAb-quantum dot conjugates (**E**) measured by dynamic light scattering (Malvern Zetasizer). Zeta potential of quantum dots (**F**) and mAb-quantum dot conjugates (**G**) determined using a Malvern Zetasizer.

The emission spectra of QDs and antibody-coupled QDs were obtained using a multifunctional enzyme marker with a fixed excitation wavelength of 450 nm. The maximum emission wavelengths were 605 and 625 nm for QDs and antibody-labeled QDs, respectively (Fig. 4A). This red shift may be attributed to the quantum size effect, reduced nonradiative transition probability, enhanced dipole interaction, and increased Stokes shift. The unchanged full width at half maximum of QDs-mAb suggests no aggregation or irregular distribution. Agarose gel electrophoresis and SDS-PAGE confirmed the larger molecular weight of QDs-mAb compared to QDs (Fig. 4B and C). DLS analysis revealed hydrated sizes of 205.7 and 23.1 nm for QDs-mAb and QDs, respectively, with good dispersibility (PDI: 0.232 and 0.216) (Fig. 4D and E). The zeta potentials were 12.5 mV for QDs-mAb and 21.2 mV for QDs (Fig. 4F and G). These results demonstrate the successful preparation of QDs-mAb, with the covalent bond ensuring stability during storage at 4℃ for up to 1 month.

### Optimization of QDs-B-ELISA conditions

**Fig 5:**
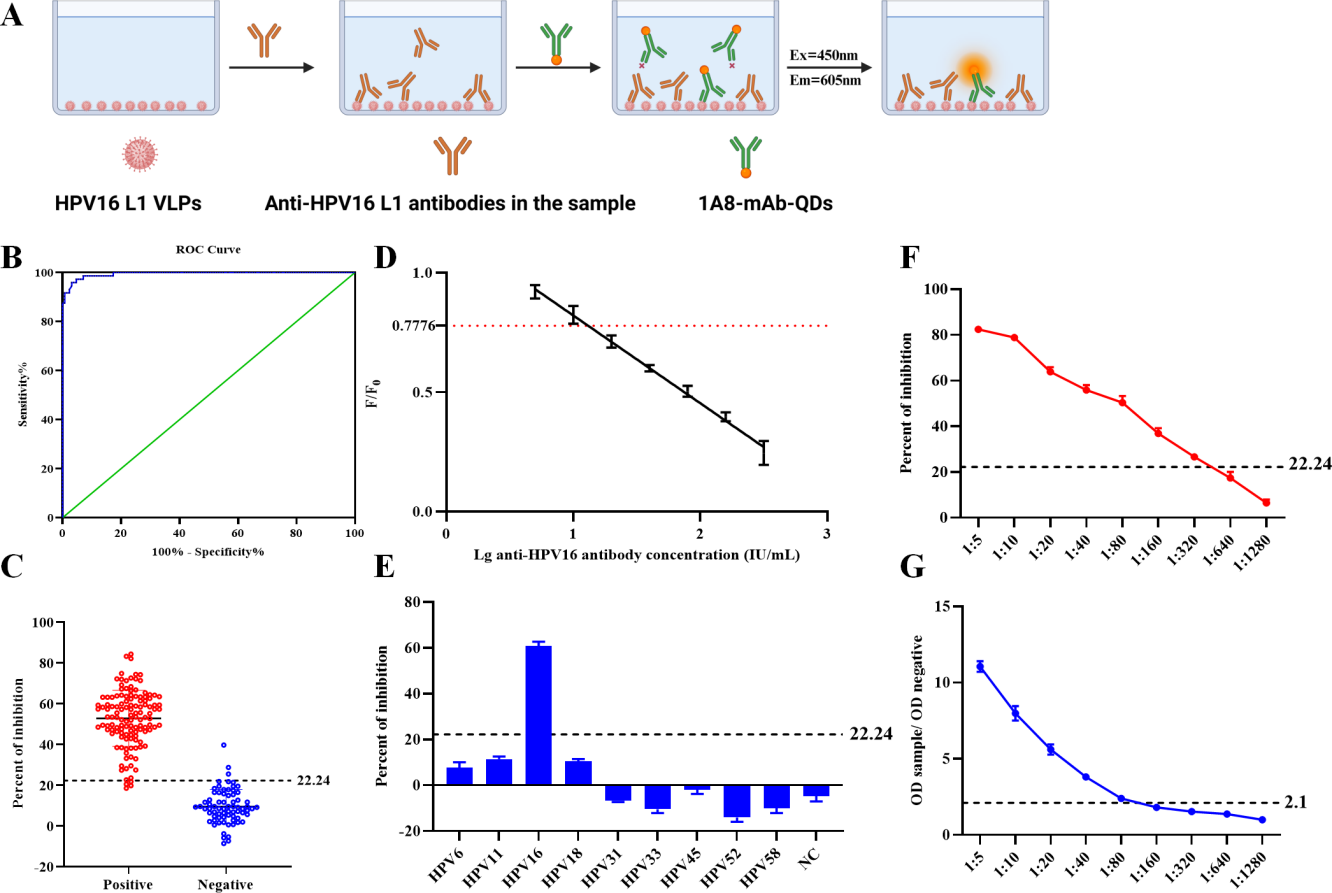
Establishment and optimization of the QDs-B-ELISA method. (**A**) Schematic representation of the QDs-B-ELISA method principle. (**B**) ROC curve analysis. Based on QDs-mAb 1A8, the area under the curve is 0.9945 (95% confidence interval: 0.9882–1.0). (**C**) Interactive dot plot analysis for QDs-B-ELISA. The *y*-axis represents the PI values of different serum samples. Based on QDs-mAb 1A8, QDs-B-ELISA achieves a diagnostic sensitivity of 95.83% and specificity of 96.85% at a cutoff value of 22.24%. (**D**) The quantitative detection curve for anti-HPV16-L1 antibodies in serum samples yielded a linear equation of *Y* = −0.3673*X* + 1.188, with a coefficient of determination (***R*^2^**) of 0.9872. (**E**) Analysis of the specificity of QDs-B-ELISA. (**F**) Analysis of the sensitivity of QDs-B-ELISA. (**G**) Analysis of the sensitivity of the Lsbio ELISA kit (catalog number LS-F10262-1).

Figure 5A illustrates the established quantum dot-labeled blocking ELISA. The optimal conditions were determined by optimizing the coating concentration, coating time, blocking time, serum dilution, serum incubation time, and QDs-monoclonal antibody incubation time. The optimized parameters were as follows: 800-fold dilution of HPV16-L1 VLPs (2 ng/µL), 50-fold dilution of QDs-1A8 mAb (0.87 µg/mL) (Table 3), 45 min of skim milk blocking, 80-fold serum dilution, and 1 h incubation times for both serum and QDs-mAb. Under these optimized conditions, the ratio of negative to positive serum (N/P) was maximized.

**TABLE 3:** Determination of the optimal coating concentration and probe dilution using the checkerboard titration method

QDs-1A8 mAb dilution	Ag concentration			
	0.5 µg/mL	1 µg/mL	2 µg/mL^*a*^	4 µg/mL
**1:50^*a*^**	35,832	43,752	**57,321^*a*^**	58,256
1:100	33,845	37,143	48,641	50,942
1:200	29,721	31,596	39,630	41,539
NC	23,742	24,612	25,376	2,642

^*a*^ Bold values in the table represents the optimal conditions tested.

### Standardization and determining the cutoff value and quantitative detection range for QDs-B-ELISA

After optimizing the QDs-B-ELISA protocol, 199 serum samples (127 HPV16 antibody positive and 72 HPV16 antibody negative) were tested in duplicate to evaluate assay performance. The percentage inhibition value was calculated for each sample, and statistical analysis of the ROC curves, cutoff values, diagnostic sensitivity, and specificity was performed (Fig. 5B). An interactive dot plot displaying the blocking values for these samples is presented in Fig. 5C. The area under the curve of 1 indicates perfect testing, while an AUC above 0.9 signifies high accuracy. The established test’s AUC was 0.9945 (95% CI: 99.82%–100%). With a cutoff value of 22.24%, the diagnostic sensitivity and specificity were 95.83% (95% CI: 88.45–98.86) and 96.85% (95% CI: 92.18%–98.77%), respectively. For HPV16-positive patients, the quantitative detection range of anti-HPV16-L1 antibodies in venous serum was 13–1,737.8 IU/mL (PI: 22.24%–100%). The IU values were determined using a standard curve generated with a reference standard of known HPV16-L1 antibody concentrations, provided by the CNIFDC. The antibody concentrations in the samples were calculated based on this standard curve and expressed in IU/mL (Fig. 5D).

### Evaluation of the specificity and reproducibility of the QDs-B-ELISA

To assess the specificity of the developed QDs-B-ELISA, we tested eight additional HPV subtypes (HPV6, 11, 18, 31, 33, 45, 52, and 58). All sera yielded negative results with blocking values well below the critical threshold, demonstrating the satisfactory analytical specificity of the established QDs-B-ELISA (Fig. 5E). The positive standard serum was detectable at a maximum dilution of 1:320 using this ELISA (Fig. 5F), significantly surpassing the Human Anti-HPV16-L1 antibody (IgG) ELISA Kit (catalog number LS-F10262-1) by Lsbio company, which had a maximum dilution of 1:80 (Fig. 5G). Reproducibility is crucial for determining the reliability of an experiment or study. We selected 10 serum samples (five positive and five negative) for the QDs-B-ELISA assay and calculated the coefficient of variation (CV = SD/mean × 100%) to evaluate intra- and inter-batch differences. A CV < 10% is considered sufficiently reproducible. Our study revealed intra-batch CVs ranging from 1.4% to 6.3% and inter-batch CVs from 3.6% to 9.8%, indicating that the HPV16-L1-based QDs-B-ELISA is highly reproducible (Table 4).

**TABLE 4:** The reproducibility and stability assays of the QDs-B-ELISA (*n* = 5)^*a*^

Serum	Number	M ± SD^*b*^	CV (intra-batch, %)	M ± SD^*b*^	CV (inter-batch, %)
HPV16 antibody-positive serum	1^*c*^	73% ± 3%	4.1	69% ± 4%	5.8
	2	65% ± 3%	4.6	65% ± 4%	6.2
	3	64% ± 4%	6.3	62% ± 4%	6.5
	4	54% ± 3%	5.6	51% ± 3%	5.9
	5	43% ± 2%	4.7	45% ± 4%	8.9
HPV16 antibody-negative serum	6^*d*^	9% ± 0.3%	3.3	11% ± 0.4%	3.6
	7	8% ± 0.3%	3.8	7% ± 0.4%	5.7
	8	12% ± 0.3%	2.5	9% ± 0.4%	4.4
	9	14% ± 0.2%	1.4	5% ± 0.2%	4
	10	5% ± 0.1%	2	4% ± 0.3%	7.5
After 30 days					
HPV16 antibody-positive serum	1^*c*^	67% ± 4%	6	79% ± 4%	5
	2	45% ± 2%	4.4	46% ± 2%	4.3
	3	55% ± 2%	3.6	51% ± 5%	9.8
	4	49% ± 3%	6.1	48% ± 3%	6.3
	5	59% ± 1%	1.7	63% ± 3%	4.8
HPV16 antibody-negative serum	6^*d*^	8% ± 0.2%	2.5	13% ± 1%	7.7
	7	6%±0.2%	3.3	8% ± 0.3%	3.8
	8	13% ± 0.4%	3.1	8% ± 0.4%	5
	9	16% ± 0.6%	3.8	6% ± 0.3%	5
	10	7% ± 0.3%	4.3	6% ± 0.4%	6.7

^*a*^ All the above experimental results were obtained in five independent experiments.

^*b*^ M,mean of percentage of inhibition; SD: standard deviation of percentage of inhibition.

^*c*^ 1, anti-HPV type 16 standard positive serum.

^*d*^ 6, anti-HPV type 16 standard negative serum.

### Preliminary application of QDs-B-ELISA in the laboratory

The QDs-B-ELISA and the Human Anti-HPV16-L1 antibody (IgG) ELISA Kit (catalog number LS-F10262-1) by Lsbio company were used to analyze 170 unknown human serum samples from Henan Cancer Hospital. The QDs-B-ELISA identified 123 HPV16 antibody-positive cases and 47 negative cases, while the Lsbio ELISA kit detected 124 positive cases and 46 negative cases. The concordance rate between the QDs-B-ELISA and the commercial Lsbio ELISA kit was 97.06% ([121 + 44]/170), demonstrating good consistency (kappa = 0.8893) (Table 5). The data revealed no significant difference between the QDs-B-ELISA and the commercial kit.

**TABLE 5:** Detection of anti-HPV16-L1 antibodies in human serum samples

		B-ELISA			
	Serum samples	Positive sample No.	Negative sample No.	Total	Coincidence
Lsbio (catalog number LS-F10262-1)	Positive sample number	121	3	124	
	Negative sample number	2	44	46	
	Total	123	47	170	97.06%
	Kappa value			0.8893	

## DISCUSSION

HPV is strongly associated with various malignancies, including cervical cancer (30). Three prophylactic vaccines—Gardasil 9, Gardasil, and Cervarix—induce neutralizing antibodies against HPV16 and HPV18, providing effective protection against infection (31, 32). However, reliable, rapid, and accessible methods for evaluating immune responses and vaccine durability remain limited, particularly in low-resource settings (https://www.hpvcentre.net/statistics/reports/XWX.pdf). Given the limited availability of accessible immune monitoring tools, detecting blocking antibodies against L1 proteins from high-risk HPV types is essential for evaluating vaccine efficacy and optimizing immunization strategies.

In this study, we developed a QDs-B-ELISA to quantitatively detect anti-HPV16-L1 antibodies using HPV16-L1 VLPs as the coated antigen. This method demonstrated high sensitivity and specificity with minimal cross-reactivity, making it a valuable tool for large-scale seroepidemiological studies. While nucleic acid-based assays confirm active infections by detecting viral DNA, serological assays like B-ELISAs offer complementary value by revealing the immune response against viral proteins.

Our QDs-B-ELISA improves upon conventional ELISA techniques by incorporating quantum dots as fluorescent probes, which enhance sensitivity, broaden the dynamic range, and improve photostability. This platform allows for reliable detection of low antibody concentrations, which could be instrumental for monitoring early infections and assessing long-term immunity following vaccination. In comparison to assays that rely on organic fluorescent dyes or enzyme-based detection systems, QDs reduce the risk of signal degradation, further improving diagnostic accuracy and reproducibility.

One of the key findings in our study is the potential application of the QDs-B-ELISA in both clinical and research settings. By enabling the quantification of anti-HPV16-L1 antibody levels, this assay could serve as a valuable biomarker for tracking the progression of HPV16 infections and evaluating the effectiveness of therapeutic interventions (33). Moreover, it could play an important role in vaccine development, particularly in assessing the strength and duration of immune responses induced by HPV vaccines (34). This is especially relevant for long-term monitoring of vaccinated individuals, where traditional serological assays may lack the sensitivity to detect declining antibody levels over time.

Existing HPV serological assays, such as cLIA, VLP-MIA, and PBNAs, have been widely used by research groups around the world for detecting neutralizing antibodies against HPV (7–10). These methods are well-established and have been instrumental in evaluating vaccine efficacy. Each method, however, has its own strengths and limitations. For example, cLIA and VLP-MIA are highly sensitive and allow for multiplexing, but they often require complex instrumentation and technical expertise, limiting their accessibility in low-resource settings. High-throughput PBNAs, while highly accurate, can be time-consuming and labor-intensive. Thus, the QDs-B-ELISA offers a valuable addition to the existing arsenal of HPV serological assays by balancing sensitivity, accessibility, and cost-effectiveness.

Our QDs-B-ELISA provides a complementary approach to these methods, offering a balance between sensitivity, ease of use, and cost-effectiveness. By leveraging the unique optical properties of quantum dots, our assay achieves a lower detection limit and a broader dynamic range than traditional ELISAs, without the need for sophisticated equipment. This makes it a promising alternative for large-scale screening and surveillance, particularly in settings where resources are limited, but there is a need for highly sensitive and specific diagnostic tools.

Despite its advantages, our study has several limitations. The small sample size necessitates further validation with larger cohorts to confirm the robustness of the assay across different populations. Additionally, this study was cross-sectional, capturing antibody levels at a single point in time. Future research should involve longitudinal studies to track changes in anti-HPV16-L1 antibody levels, particularly in vaccinated individuals.

Optimizing the assay for high-throughput automation is another area for improvement. Standardizing the protocol for clinical laboratories will require additional efforts to ensure reproducibility and streamline processing. Expanding the assay to detect antibodies against other high-risk HPV types, such as HPV18, would also enhance its versatility for broader applications in HPV monitoring and vaccine evaluation.

### Conclusion

In conclusion, our study presents a new and reliable HPV16 QDs-B-ELISA assay for detecting anti-HPV16-L1 antibodies in human serum. Despite limitations, its high sensitivity, specificity, and potential clinical applications highlight the significance of further exploring and optimizing this assay. The development of this method contributes to the ongoing efforts in HPV research, potentially leading to improved diagnostic tools, vaccine efficacy monitoring, and disease management strategies.

## Data Availability

All raw data can be made available upon reasonable request.
